# Supplementary material for: Optimization of Zebrafish Larvae Sectioning for Mass Spectrometry Imaging
Source: Pharmaceuticals (Basel). 2022 Oct 7;15(10):1230. doi: 10.3390/ph15101230 (PMC9608760; doi:10.3390/ph15101230)
Supplement: Supplementary file 1 [file pharmaceuticals-15-01230-s001.zip › pharmaceuticals-1909103-supplementary.pdf]

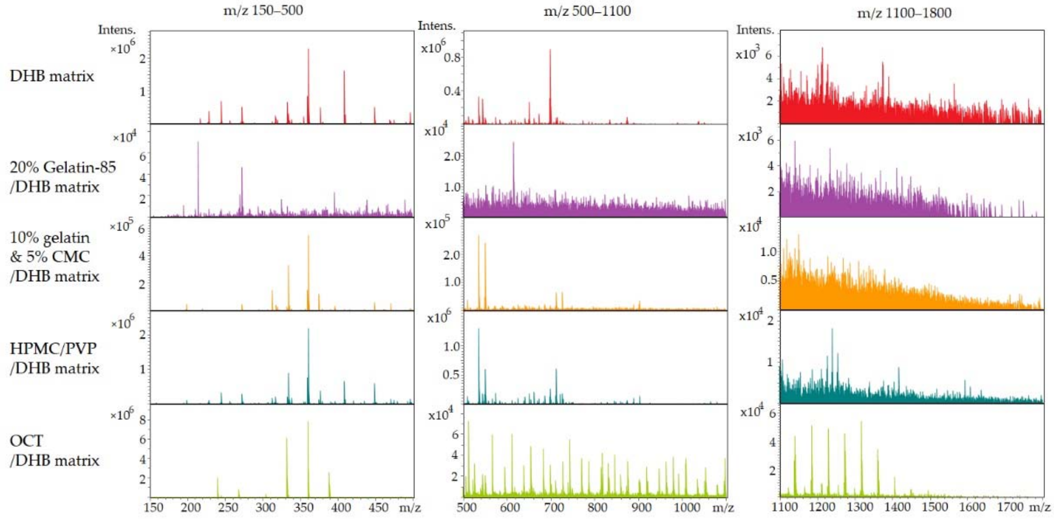

Figure S1. Comparison of mass spectra of different embedding media under MALDI conditions with DHB matrix coating at three m/z windows: m/z 150-500; m/z 500-1100; m/z 1100-1800 (each spectrum is scaled to the highest peak of its own mass window)

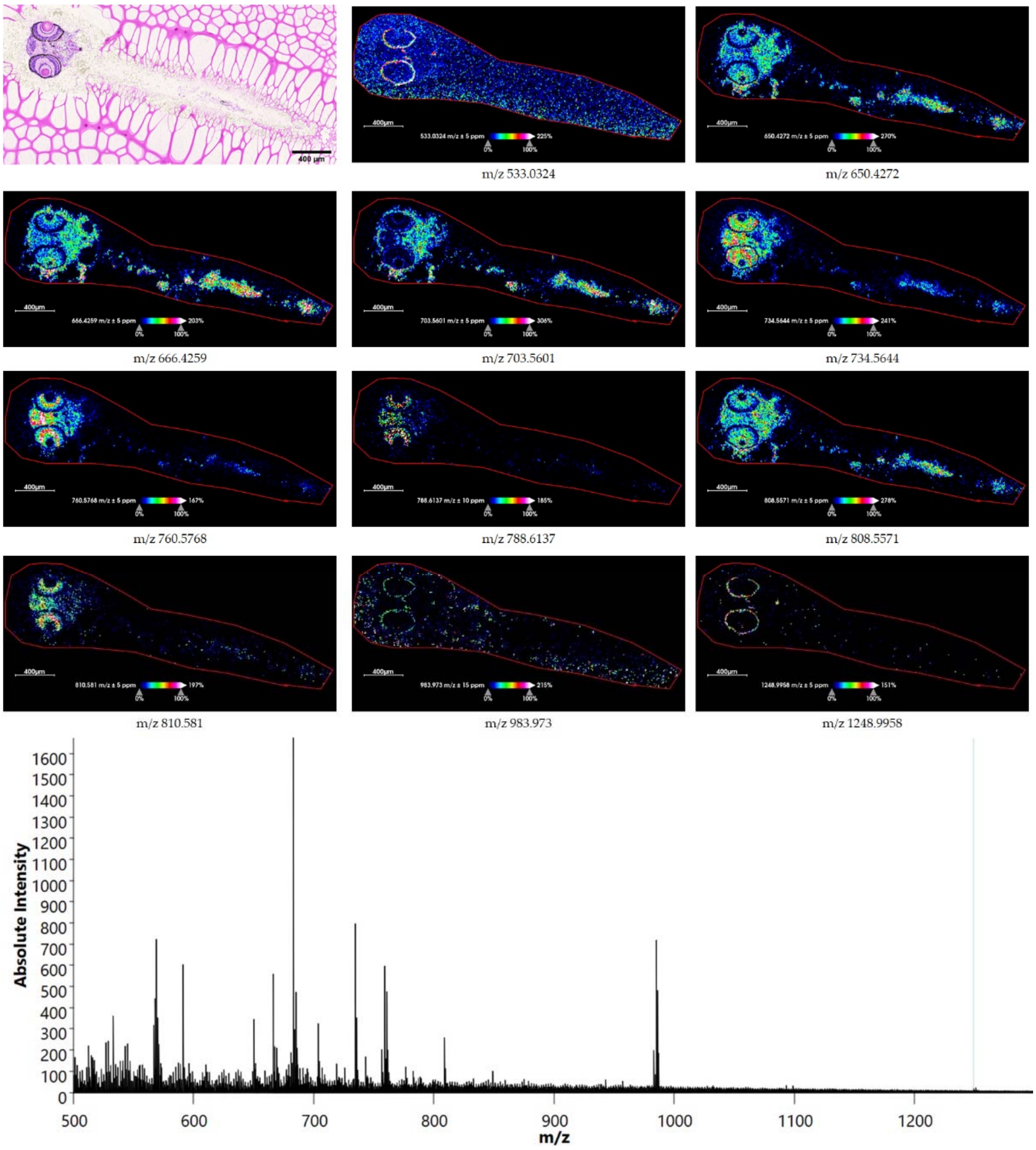

Figure S2 Mass spectrometry imaging of a zebrafish larva section in m/z range 500-1300. Top: ion images of selected ions with distinct pattern matching well with H&E optical image, spatial resolution for ion images is 10 μm; bottom: average mass spectrum of this imaging session.

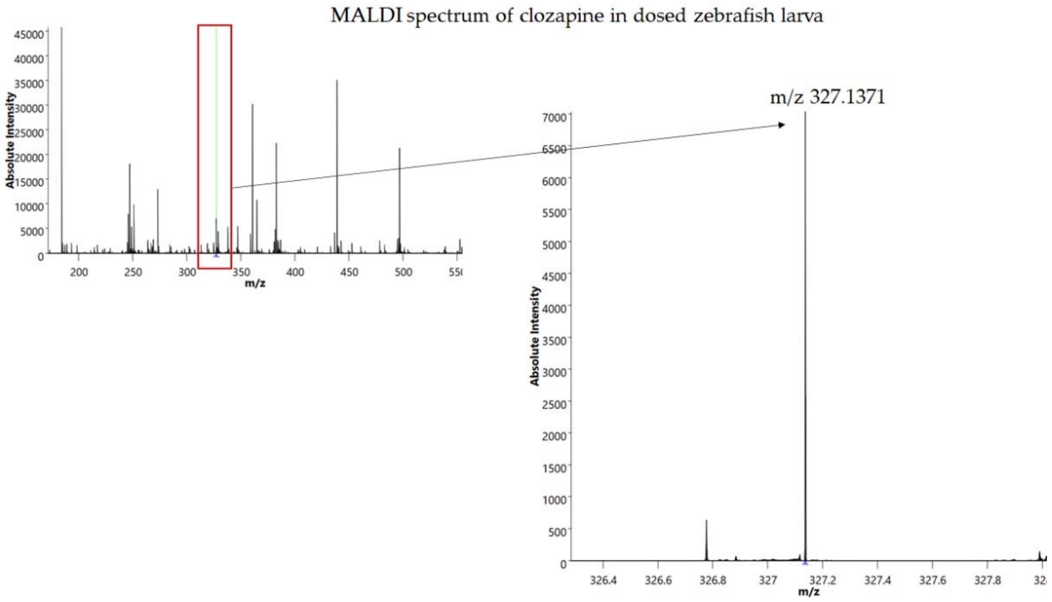

Figure S3 MALDI spectrum of clozapine in dosed zebrafish larva. The ion image is in the main text (Figure 5b left)

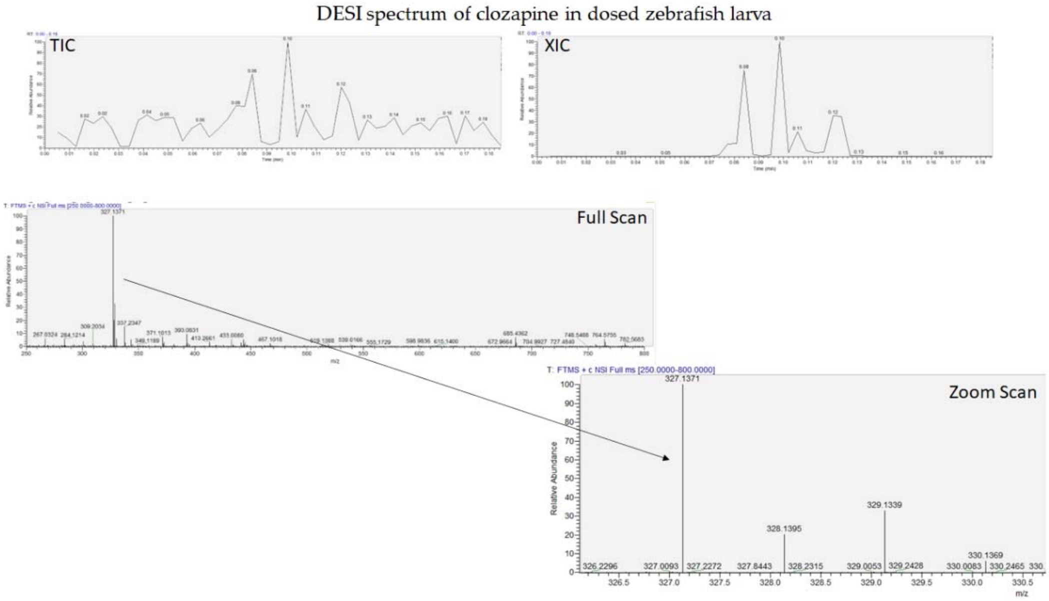

Figure S4 DESI spectrum of clozapine in dosed zebrafish larva. The ion image is in the main text (Figure 5b right)
